# Prediction of miscarriage in first trimester by serum estradiol, progesterone and β-human chorionic gonadotropin within 9 weeks of gestation

**DOI:** 10.1186/s12884-021-04158-w

**Published:** 2022-02-10

**Authors:** Wenhui Deng, Rui Sun, Jun Du, Xue Wu, Lijie Ma, Min Wang, Qiubo Lv

**Affiliations:** 1 Department of Obstetrics and Gynecology, Beijing Hospital, National Center of Gerontology; Institute of Geriatric Medicine, Chinese Academy of Medical Sciences, Beijing, 100730 P. R. China; 2 Department of Obstetrics and Gynecology, Tongzhou Maternal and Child Health Hospital of Beijing, Beijing, 101100 P. R. China; 3 Department of Pathology, Beijing Hospital, National Center of Gerontology; Institute of Geriatric Medicine, Chinese Academy of Medical Sciences, Beijing, 100730 P. R. China

**Keywords:** Pregnancy, Estradiol, Progesterone, β-HCG, Miscarriage

## Abstract

**Purpose:**

To predict miscarriage outcome within 12 weeks of gestational age by evaluating values of serum estradiol, progesterone and β-human chorionic gonadotropin (β-HCG) within 9 weeks of gestation.

**Methods:**

One hundred sixty-five women with singleton pregnancies were retrospectively studied. Estradiol, progesterone and β-HCG levels were measured at 5–6 weeks of gestation and the measurements were repeated at 7–9 weeks. According to pregnancy outcome at 12 weeks of gestation, 71 cases were categorized into miscarriage group, and 94 cases into group of normal pregnancy. Each group was further divided into 5–6 and 7–9 weeks of gestation sub-group. Predictive values of estradiol, progesterone and β- HCG levels at 5–6 weeks and 7–9 weeks of gestation were analyzed with receiver operating characteristic (ROC) curves and logistic regression.

**Results:**

Serum levels of estradiol at 7–9 weeks identified miscarriage with an area under the ROC curve (AUC) of 0.866 (95% CI 0. 793 ~ 0.938, *P* = 0.000), diagnostic cutoff value of 576 pg/ml, sensitivity of 0.804, and specificity of 0.829 respectively at the optimal threshold, according to Youden index. Progesterone levels at 7–9 weeks were with AUC of 0.766 (95% CI 0. 672 ~ 0.861, *P* = 0.000), cutoff value of 15.27 ng/ml, sensitivity of 0.921, and specificity of 0.558, respectively; Estradiol at 5–6 weeks were with AUC of 0.709 (95% CI 0. 616 ~ 0.801, *P* < 0.001), the diagnostic cutoff value of 320 pg/ml, sensitivity of 0.800, and specificity of 0.574, respectively.

The performance of the dual markers of estradiol and progesterone analysis (AUC 0.871, CI 0.793–0.950), three-markers analysis (AUC 0.869, CI 0.759–0.980)were slightly better than the single marker at 7-9 weeks. β-HCG or progesterone provide additional utility of estradiol prediction at 5–6 weeks with AUC 0.770 (0.672–0.869) for β-HCG and estradiol, AUC0.768(CI 0.670–0.866) for β-HCG, estradiol and progesterone and AUC 0.739 (CI 0.651–0.827) for progesterone and estradiol.

**Conclusions:**

Low serum levels such as dual of estradiol and progesterone or estradiol alone at 7–9 weeks, β-HCG or progesterone combing estradiol at 5–6 weeks of gestation can be used better to predict miscarriage in first trimester.

## Introduction

Pregnancy loss occurs in approximately 15–25% of pregnancies [1]. It is still the most common pregnancy complication affecting women’s physical and mental health. Repeated Clinical examinations and treatments for threatened abortion also leads to economic and mental burdens on patients. But there is no reliable clinical indicator to predict it early yet. Low values and low growth rates of estradiol and β-human chorionic gonadotropin (β-HCG) probably indicate bad pregnancy outcome [2].

HCG - one of the most important endocrine factors as we known, can inhibit T cell stimulation and avoid variant stimulation of T lymphocyte reaction of embryo damage [3]. Initial HCG, HCG ratio [4], and too low rising speed of HCG at early pregnancy may indicate miscarriage or ectopic pregnancy [2]. Recent researches suggested that serum progesterone [5, 6], estradiol [2] and ultrasound [7, 8] can identify women with miscarriage. Whereas one study suggest that the most commonly used biomarkers of serum HCG and progesterone are not useful in predicting outcome of a pregnancy with a viable fetus [9]. The factors that predict pregnancy losses are not well understood [8]. Although both ultrasound and serial HCG values can, to some extent in the clinic, identify women at risk of miscarriage, study remains sparse on other more effective biomarkers, to identify at-risk pregnancies, especially before the onset of clinical symptoms [10].

Estrogen stimulating endometrial hyperplasia and myometrium thickness, increases blood supply and enhances uterine contraction force. Studies suggest that estradiol be an important factor to maintain early pregnancy [11, 12]. In the 4 to 8 weeks of pregnancy, serum estradiol level was positively correlated with gestational age. Estradiol level at early pregnancy can reflect the quality of the dominant follicle and the function of corpus luteum as well as help maintain corpus luteum [13, 14]. Serum estradiol were significantly lower in pregnant women with abortion than in those with normal pregnancy [13]. Estradiol and immune cells in pregnant women can work together to maintain normal pregnancy [12]. Estradiol has not been used as widely as β-HCG and P, and its value in predicting pregnancy outcome is unclear [2].

Since endocrine factors, such as HCG, progesterone and estrogen, are critical for normal pregnancy, low levels of them may be markers of miscarriage. Retrospective analysis was carried out on the data of pregnant women diagnosed and followed at Obstetrics and Gynecology Clinic of Beijing Hospital from July 2015 to July 2016 and predicted miscarriage within the 12 weeks of gestation with receiver operating characteristic (ROC) curve of serum estradiol, progesterone and β-HCG at 5–9 weeks of gestation.

## Methods

### Study population

**Fig. 1:**
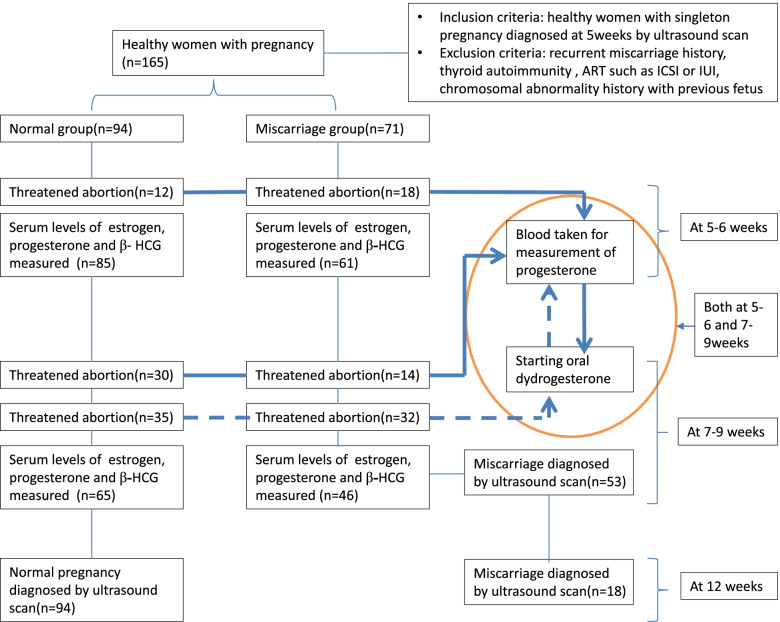
Flow chart of study

One hundred sixty-five women with singleton pregnancies were retrospectively studied. The healthy women aged between 21 and 40, with 5 weeks of singleton pregnancy were diagnosed at first visit by ultrasound screening (Ultrasonic apparatus GE V730). Their data was analyzed from July 2015 to July 2016 at Obstetrics and Gynecology Clinic of Beijing Hospital. Patients with recurrent miscarriage history, thyroid autoimmunity were excluded from the study. Pregnancies achieved by artificial assisted pregnancy technology (ART) such as intracytoplasmic injection (ICSI) or intrauterine insemination (IUI) was excluded also. The pregnancy was the first to the third time of women and there was no chromosomal abnormality history with previous fetus. Serum estradiol, progesterone and β-HCG levels of these women were measured twice at 5–6 weeks and 7–9 weeks of gestational age respectively. The research flow chart is shown in Fig. 1.

### Study design and setting

Seventy-one cases diagnosed as miscarriage within 12 weeks of pregnancy were categorized into miscarriage group and the other 94 cases at 12 weeks were into normal pregnancy group (Table 1). Each group was further divided into 5–6 weeks sub-group and 7–9 weeks sub-group. Threatened abortion was diagnosed with vaginal bleeding or abdominal pain occurrences and treated with oral dydrogesterone 20 mg twice a day. Eighteen cases in miscarriage group and 12 cases in normal group were diagnosed with threatened abortion at 5-6 weeks. Their blood was taken for measurement of progesterone and then they were treated with dydrogesterone (Fig. 1 and Table 2). At 7–9 weeks, totally 65 cases were diagnosed with threatened abortion in normal group and 71 cases in miscarriage group. The blood taken for measurement of progesterone was prior to or after starting dydrogesterone treatment at 7-9 weeks (Fig.1 and Table 3).

**Table 1:** The women's age, number of pregnancy and parity in two groups

Groups	Number of cases	Age (years old)	Cases with number of pregnancy			Cases with parity	
			1	2	3	0	1
Normal pregnancy	94	34.7± 6.7	52	37	5	90	4
Miscarriage	71	32.5± 8.9	41	23	7	69	2
*P*		0.07#	0.440#, a			0.481#, a	

# No significantly different between miscarriage and normal pregnancy

a Chi-Square test

**Table 2:** Progesterone levels before starting oral dydrogesterone treatment in women with or without threatened abortion at 5-6 weeks of gestation in normal and miscarriage groups

Groups (number of cases)	Progesterone levels (ng/ml) before starting oral dydrogesterone treatment in women with threatened abortion (number of cases)	Progesterone levels (ng/ml) in women without threatened abortion and oral dydrogesterone treatment (number of cases)	*P*
Normal pregnancy (*n*=85)	22.56±7.40 (*n*=12)	25.62±5.80 (*n*=73)	0.107#
Miscarriage (*n*=61)	23.26±8.23 (*n*=18)	22.17±5.60 (*n*=43)	0.550#

# No significantly different between women with and without threatened abortion in miscarriage and normal pregnancy

**Table 3:** Progesterone levels after or before starting oral dydrogesterone treatment at 7-9 weeks of gestation in women with threatened abortion in normal and miscarriage groups

Women with threatened abortion and treated with oral dydrogesterone in two groups (number of cases)	Progesterone levels (ng /ml) after oral dydrogesterone treatment (number of cases)	Progesterone levels (ng /ml) before starting oral dydrogesterone treatment(number of cases)	*P*
Normal pregnancy (*n*=65)	26.12±7.18 (*n*=35)	23.57±7.20 (*n*=30)	0.159#
Miscarriage (*n*=46)	20.12±6.31 (*n*=32)	17.18± 6.18 (*n*=14)	0.151#

# No significantly different between after and before starting oral dydrogesterone treatment in miscarriage and normal pregnancy

Transvaginal ultrasound scans were performed by the same experienced attending doctor. Normal pregnancy group was diagnosed by ultrasound screening (Ultrasonic apparatus GE V730) with fetus survival in accordance with 12 weeks of gestational age. Miscarriage was diagnosed with ultrasonic criteria [15]: 1) Crown–rump length of ≥7 mm and no heartbeat; 2) Mean sac diameter of ≥25 mm and no embryo; 3) Absence of embryo with heartbeat ≥2 weeks after a scan that showed a gestational sac without a yolk sac; 4) Absence of embryo with heartbeat ≥11 days after a scan that showed a gestational sac with a yolk sac. The number of patients who were performed ultrasound scans at different weeks is shown in Fig. 1.

### Estradiol, progesterone and β-HCG measurement

Serum estradiol, progesterone and β-HCG levels were measured with chemiluminescence on Roche Elecsys 2010 type instrument. The variations were 6.4% between the batches and 6.0% within the batches.

### Statistical analysis

The statistical analysis was performed using SPSS 16.0 software. A *P-*value of ≤0.05 was considered statistically significant for all statistical tests. In order to obtain measurement data for single factor variance analysis, the comparison between groups was conducted by Tukey post hoc test. The Chi-square test was adopted to show the number of pregnancy and parity significance compared between two groups. The miscarriage outcome, as state variables, was calculated based on the area under ROC curve (AUC) of estradiol, progesterone and β-HCG levels, respectively. The Youden index was used to determine the optimal sensitivity and specificity; cutoff values to Youden index’s biggest diagnosis were calculated, which were the degree of sensitivity, specific, and missed diagnosis to this point. Diagnosis agreements between estradiol, progesterone, β-HCG cutoff levels and ultrasound were obtained with using the kappa coefficient (κ). Univariate and multivariate logistic regressions were also used for the further measurements.

## Results

### Levels of serum estradiol, progesterone and β- HCG

Characteristics of the two groups are presented in Table 1. The two groups were similar in women’s age, number of pregnancies and the parity. Only three women with miscarriage were willing to detect chromosomal karyotype of the embryos. The results were normal. Table 2 presents the progesterone levels before starting oral dydrogesterone treatment in women with or no threatened abortion at 5–6 weeks of gestation in normal pregnancy and miscarriage groups. Progesterone levels were slightly higher in normal pregnancies without giving dydrogesterone than with preparing to take oral dydrogesterone (Table 2). Table 3 presents the blood taken for measurement of progesterone before or after starting dydrogesterone treatment at 7–9 weeks of gestation in threatened abortion women of two groups. It showed higher levels of progesterone with giving dydrogesterone than without starting oral dydrogesterone in two groups. But the progesterone levels were not significantly different between without oral dydrogesterone and with oral dydrogesterone in two groups (Tables 2 and 3).

The mean maternal age in miscarriage was not significantly (*P* > 0.05) different from those in the normal pregnancy group at 5 - 6 weeks or 7–9 weeks of gestation (Tables 4 and 5).

**Table 4:** Age, serum levels of estradiol, progesterone and β-HCG in normal pregnancy and miscarriage groups at 5-6 weeks of gestation by Tukey post hoc test

Group	The number of cases	Age (years old)	Estradiol (pg /ml)	Progesterone (ng /ml)	β-HCG (mIU/ml)
Normal pregnancy	85	31.5± 6.8	559±356	22.69±7.87	5295.98a
Miscarriage	61	32.3± 3.7	375±233	22.07±9.58	2926.25a
*P*		0.132#	0.001**	0.684#	0.063#

**Significantly lower in miscarriage than that in normal pregnancy (*P*<0.01)

# No significantly different between miscarriage and normal pregnancy

a For the geometric mean

**Table 5:** Age, serum levels of estradiol, progesterone and β-HCG in normal pregnancy and miscarriage groups at 7 to 9 weeks of gestation by Tukey post hoc test

Group	The number of cases	Age (years old)	Estradiol (pg/ml)	Progesterone (ng/ml)	β-HCG (mIU/ml)
Normal pregnancy	65	34.1±6.2	1209±785	25.84±8.61	32549a
Miscarriage	46	32.2±3.7	39±289	17.15±8.62	17700a
*P*		0.342#	0.000**	0.000**	0.060#

**Significantly lower in miscarriage than that in normal pregnancy (*P*<0.01);

# No significantly different between miscarriage and normal pregnancy;

a For the geometric mean

At the 5–6 weeks and 7–9 weeks of gestation, serum estradiol levels were lower in miscarriage group as compared with those in normal pregnancy group (*P* < 0. 05) (Table 4 and 5). Similarly, patients with miscarriage showed lower progesterone levels at 7 - 9 weeks of gestation (*P* < 0. 05) (Table 5). In contrast, no statical differences were observed between miscarriage and normal pregnancy groups in serum progesterone concentration at 5–6 weeks of gestation and β-HCG levels at 5–6 weeks and 7–9 weeks of gestation, though the levels were higher in normal groups (*P* > 0.05) (Table 4 and 5).

Normal pregnancy group had significantly elevated serum levels of estradiol, progesterone and β-HCG at 7–9 gestational weeks as compared with those at 5–6 gestational weeks (*P* < 0. 05) (Table 6).

**Table 6:** Serum levels of estradiol, progesterone and β-HCG at different gestational ages in normal pregnancy groups

Gestational age	Number of cases	Estradiol (pg/ml)	Progesterone (ng/ml)	β-HCG (mIU/ml)
5-6 weeks	85	559±356	22.69±7.87	5295.98a
7-9 weeks	65	1209±785	25.84±8.61	32549a
*P*		0.000**	0.025*	0.000**

*,**Significantly (*P<0.05, **P<0.01) higher at 7-9 gestational weeks than that at 5-6 weeks in normal pregnancy women;

a For the geometric mean

### Serum estradiol, progesterone and β-HCG levels of ROC curve analysis

**Fig. 2:**
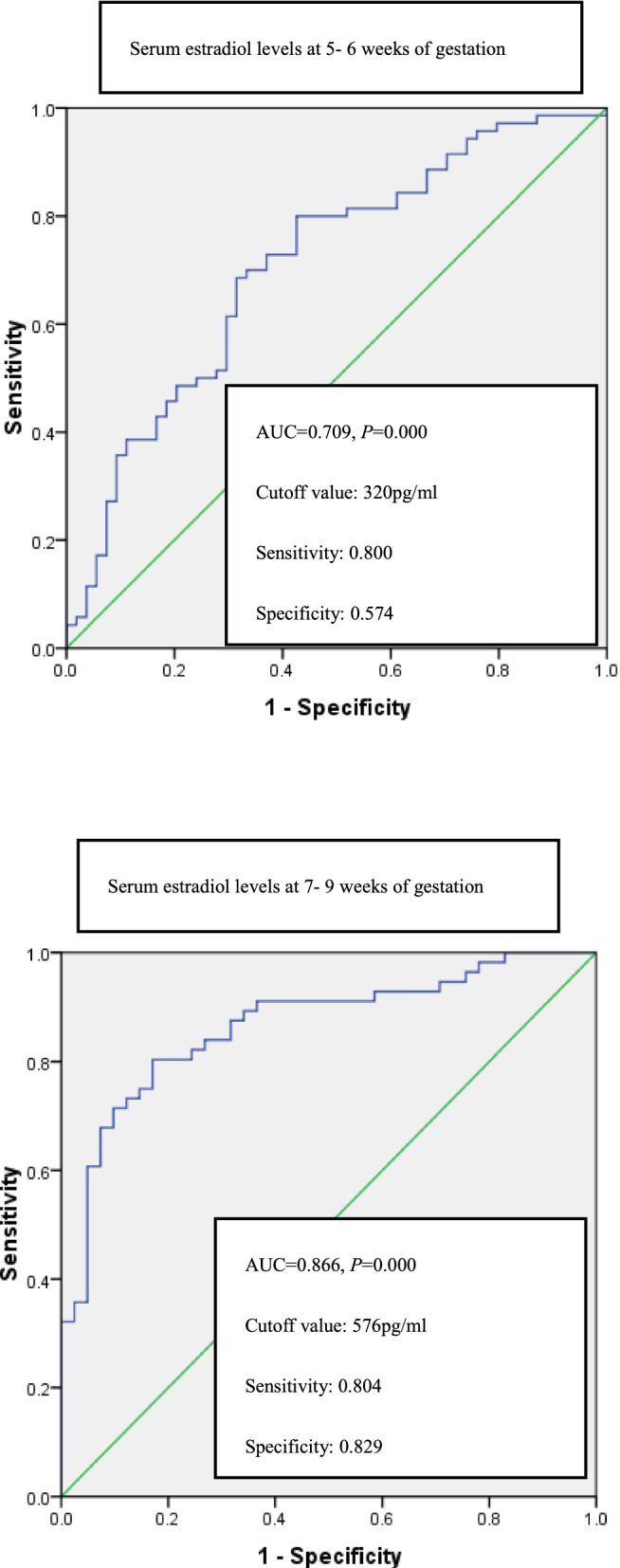
Estradiol receiver- operating characteristic (ROC) curves at 5–6 weeks and 7–9 weeks of gestation. The cutoff value, the sensitivity and specificity degrees were also shown

**Fig. 3:**
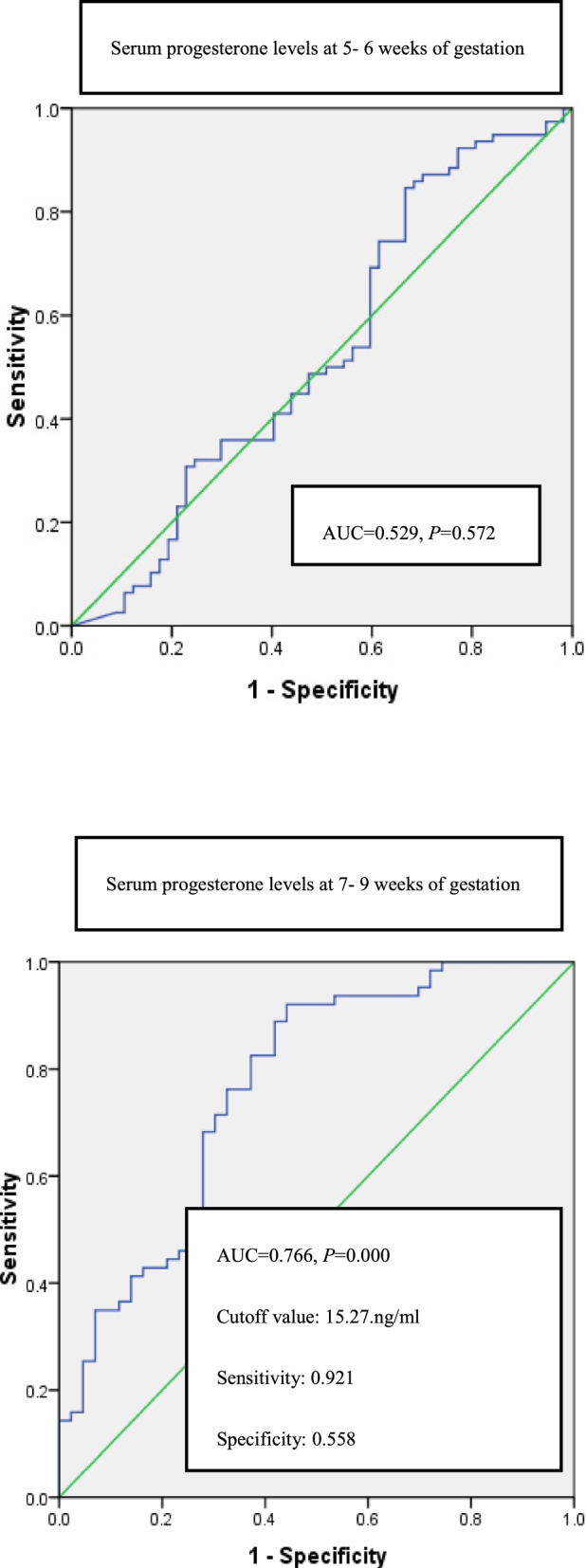
Progesterone receiver- operating characteristic (ROC) curves at 5–6 weeks and 7–9 weeks of gestation. The cutoff, the sensitivity and specificity degrees were also shown

At 7–9 weeks, AUC of serum estradiol was 0.866, with 95% confidence interval (CI) (0.793 ~ 0.938) (*P* = 0.000, Fig. 2). At the optimal threshold according to Youden index up to 0.633 for cutoff value of 576 pg/ml, the sensitivity, the specificity and the missed diagnosis degrees were 0.804, 0.829, and 0.171, respectively (Fig. 2). The AUC for progesterone at 7–9 weeks was 0.766, with 95% CI (0. 672 ~ 0.861) (*P* < 0.001, Fig. 3), and for estradiol at the 5–6 weeks decreased to 0.709, with 95% CI (0. 616 ~ 0.801) (*P* = 0.000, Fig. 2).

Cutoff value of serum estradiol at 5–6 weeks according to the Youden index of 0.374was 320 pg/ml, with the sensitivity of 0.800, specificity of 0.574 and missed diagnosis degree of 0.426, respectively (Fig. 2). At 7–9 weeks, cutoff value of progesterone was 15.27 ng/ml to the Youden index of 0.479, with the sensitivity of 0.921, the specificity of 0.558, and the missed diagnosis of 0.442, respectively (Fig. 3).

The levels of concordance on diagnosing miscarriage between the estradiol at 7–9 weeks, the estradiol at 5-6 weeks, progesterone at 7-9 weeks cutoff measurements and ultrasound was moderate (κ = 0.425, 0.419 and 0.599, *P* = 0.000, respectively).

However, at 5–6 weeks, AUC of serum progesterone of 0.529 showed no statistical significance (*P* = 0.572) to predict miscarriage in first trimester. Likewise, at 5–6 weeks and at 7–9 weeks, AUCs of serum β-HCG were 0.396 and 0.661with no statistical significance (*P* = 0.059, *P* = 0.059 respectively).

### Multi-marker ROC analysis

A multivariate logistic regression was performed to evaluate its ability to predict miscarriage based on all 3 biomarkers plus gestational age. At the optimal cutoffs for each marker and all logistic and AUC data were described in Table 7. The performance of the dual markers of estradiol and progesterone analysis (AUC = 0.871, CI 0.793–0.950) was slightly better than the single marker at 7-9 weeks. So was multi- marker analysis (AUC =0.869, CI 0.759–0.980). β- HCG by itself did not have a predictive AUC of 0.661 at 7-9 weeks, nor did it increase the potency of estradiol or estradiol combined with progesterone, although its combination of progesterone had greater AUC of 0.865 than 0.766 of progesterone alone. Similarly, the diagnosis probability of miscarriage was the highest 92.9% using markers of estradiol combination with progesterone or with progesterone and β- HCG at 7-9 weeks.

**Table 7:** The probabilities and area of ROC of multi-markers, dual of estradiol and progesterone, estradiol and progesterone using logistic regression and ROC analyze

Markers	Probabilities of miscarriage	*P*	Area of ROC	*P*	Cutoff value
E2+P+β-HCG (7-9 weeks)	92.9	0.001**	0.869(0.759-0.980)	0.000**	
**E2+P (7-9 weeks)**	**73.8**	0.000**	**0.871 (0.793-0.950)**	0.000**	
E2+β-HCG (7-9weeks)	92.9	0.005**	0.795(0.641-0.948)	0.000**	
P+β-HCG(7-9weeks)	82.1	0.000**	0.865(0.760-0.968)	0.000**	
**E2 (7-9 weeks)**	**85.7**	0.000**	**0.866 (0.793- 0.938)**	0.000**	576pg/ml
**P (7-9 weeks)**	**68.2**	0.000**	**0.766(0.672- 0.861)**	0.000**	15.27ng/ml
β-HCG(7-9weeks)	-	0.087#	0.661	0.059#	3098.765IU/L
E2+P+β-HCG (5-6weeks)	80.4	0.000**	0.768 (0.670-0.866)	0.000**	
E2+P (5-6 weeks)	80.4	0.012**	0.739(0.651-0.827)	0.000**	
**E2+β-HCG(5-6weeks)**	**86.3**	0.000**	**0.770(0.672-0.869)**	0.000**	
P+β-HCG(5-6weeks)	73.6	0.03*	0.667(0.561-0.772)	0.004**	
**E2 (5-6 weeks)**	**72.7**	0.000**	**0.709(0.616-0.801)**	0.000**	320pg/ml
P (5-6 weeks)	28.1	0.046#	0.529	0.572#	8.47ng/ml
**β-HCG (5-6 weeks)**	**-**	**1.050**#	**0.396**	0.059#	2858.6IU/L

*, **Significantly prediction utility for miscarriage (*P<0.05, **P<0.01)

# No significantly prediction utility for miscarriage

*E2* estradiol, *P* progesterone, *E2+P+ β-HCG* multi markers analysis

AUC of β-HCG at 0.396 or progesterone at 0.529 at 5-6 weeks showed no predictive effect itself; however, β-HCG or progesterone provide additional utility of estradiol prediction at 5–6 weeks, with AUC 0.770(0.672–0.869) for β-HCG and estradiol, AUC 0.768(CI 0.670–0.866) for β-HCG, estradiol and progesterone, and AUC 0.739 (CI 0.651–0.827) for estradiol and progesterone (see Table 7). Of course, all of these predictions at 5-6 weeks are less effective than the dual or multi markers predictions at 7–9 weeks.

## Discussion

Our study showed that the serum estradiol, progesterone and HCG levels increased with gestational age during 5–9 weeks, which was consistent with previous results [2, 16]. In the miscarriage group, estradiol and progesterone were significantly decreased. Previous studies also showed that the levels of these indicators were decreased, indicating a poor pregnancy outcome [2, 17]. However, the value of these indicators in predicting miscarriage is still unclear.

Our results showed that the serum markers within 9 weeks of gestation can be used to differentiate the possibilities of normal pregnancy and miscarriage in first trimester. Specifically, low serum estradiol levels at 5–6 and 7–9 weeks and progesterone at 7–9 weeks can serve as the predictors, while β-HCG levels showed no prediction effect.

Our further dual or multi markers analysis showed that the AUC of estradiol combined with progesterone could reach the largest 0.871, and estradiol alone was 0.866. β-HCG did not increase the predictive efficacy of estradiol combined with progesterone or estradiol at 7–9 weeks for miscarriage. The reason could be that the predictive effect of estradiol was too great to be affected by β-HCG. The predictive effect of dual or multi markers at 5–6 weeks was lower than that at 7–9 weeks. β-HCG or progesterone level alone had no predictive significance, but their combination with estradiol could improve the predictive effect of estradiol at 5–6 weeks. Especially, dual of β-HCG and estradiol could perform better. So lower levels of estradiol are the primary predictor of miscarriage at 5 to 9 weeks. Our results are consistent with Yang Li, etc’s report [2]. The difference is that our study objective was to predict miscarriage within 12 weeks, while Yang Li, etc’s was to exclude other adverse outcomes of normal pregnancy, such as biochemical ectopic pregnancy and miscarriage.

Progesterone can provide endocrine support. Renzo, etc. [18] reported high progesterone level has a calming effect, reducing the uterine contraction intensity and frequency, and thereby extends endometrial secretion period and coordination and embryonic development in planting window synchronization. It was found that progesterone can also improve the immune status by promoting the maternal-fetal interface CD56 + lymphocytes induced secretion of progesterone induced blocking factor (PIBF) to prevent immune rejection. Progesterone levels may help diagnose embryonic development. Puget etc. [19] reported that serum progesterone level lower than 6.2ng/ml indicated lower prompt embryonic vitality. The sensitivity, specificity to diagnose an early pregnancy loss were 20% and 100%.

In our study, we found that serum progesterone levels increased at 7–9 weeks of gestation in normal pregnancy compared to those at 5–6 weeks of gestation. Progesterone levels at 5–6 weeks could not predict miscarriage in first trimester. But at 7–9 weeks, progesterone levels can be a marker to predict miscarriage despite no more effective than serum estradiol levels at 7*–*9 weeks. More excitedly, it provides estradiol additional higher utility of prediction with the largest AUC of 0.871.

Our result showed that progesterone could be a predictor at 7-9 weeks because the onset of substantial progesterone production is the 7th gestational week [16]. Similar in other studies, high progesterone level on the day of embryo transfer was a predictor of a higher ongoing pregnancy rate [20]. In Ku CW et al’s report [5], serum progesterone concentration increased linearly with gestational age from 5 to 13 weeks in women with normal pregnancies, and women with spontaneous miscarriage showed a marginal and non-significant increase in serum progesterone. Our results also showed higher progesterone levels at 7–9 gestational weeks than 5–6 gestational weeks in normal pregnancy. Similarly, women with miscarriage showed lower progesterone levels than normal pregnancy at 7–9 weeks of gestation. The studies suggest the essential role of progesterone in supporting an early pregnancy.

Progesterone levels at 7–9 weeks lower than cutoff value of 15.27 ng/ml could predict miscarriage in our study. That is accordance with Lek SM et al’s study [6]. They also presented that the cut-off value for serum progesterone (35 nmol/L) demonstrated clinical relevance and allow clinicians to stratify patients into high and low risk groups for spontaneous miscarriage. The higher cutoff value of progesterone in our study may be because the inspection interval was 7–9 weeks to predict miscarriage within 12 weeks of gestation, while in Lek SM’s study was 6–10 weeks to predict miscarriage prior to 16 weeks of gestation.

HCG is a glycoprotein secreted after fertilization of sterol cell in 6-8 weeks. It peaks at 8–12 weeks of gestation for l - 2 weeks and then begins to reduce [21]. Low serum HCG during pregnancy is considered a natural miscarriage or a poor pregnancy [2, 4, 21]. Dynamic level of HCG may serve better because serum HCG is increased at the rate of 66% per 48 h in normal pregnancy. HCG increase rate less than 66% indicates adverse pregnancy outcomes [21, 22].

Compared with those results, β-HCG was tested at 5–9 weeks only twice in our study, which is not enough to predict the overall miscarriage outcome within 12 weeks. It may be because that pregnancy is an unstable and susceptible period [23]. However more interestingly, β-HCG combined with estradiol at 5–6 weeks significantly improved the prediction. It may be related to the HCG plateaued in this period [22].

Our results were similar to other studies which found that low values and low growth rates of estradiol and β-HCG probably indicate bad pregnancy outcomes [2]. However, another report revealed a higher, not lower, level of β**-**HCG and estrogen during the first 6 weeks of pregnancy, suggesting a novel association between β**-**HCG, estrogen, and threatened abortion [23, 24]. Unfortunately, this study was limited by its small sample size, unconvincing trial design, and inadequate exploration of the underlying mechanisms [23].

Early miscarriage is usually diagnosed with pelvic ultrasound after a woman experienced vaginal bleeding or abdominal pains [22]. If ultrasound at the 8.5 weeks pregnancy showed embryonic survival, 95% of pregnancies within 14 weeks would not end up to miscarriage [25]. Findings were controversial although some studies on ultrasound scan, including subchorionic hemorrhage, fetal heart rate, crown–rump length and yolk sac diameter associated with pregnancy loss existed [7, 8]. So, prediction markers before 8.5 weeks need to be further inspected. It was found in our study that estradiol at 5–9 weeks of gestation makes a key effect on prediction of miscarriage in first trimester.

Current findings differ from previous study [2], which showed the values of estradiol at every gestational week from 5 to 8 weeks to predict miscarriage. There are two reasons for the 5–6 weeks and 7–9 weeks groups used in our study.

First is related to the period of prenatal clinic examination. Generally, ultrasound scan may show the fetal sac at 5–6 weeks of gestation. More women are willing to come to the hospital during this period for examination. The women and doctors would test serum estradiol, progesterone and β- HCG to understand the pregnancy status. These markers were getting more commonly used in the ART pregnancy. The second period of time to the hospital for examination is 7–9 weeks when most of the fetal bud and fetal heart could be seen by ultrasound [8]. Moreover, 95% of pregnancies within 14 weeks would not end up to miscarriage if embryo survives before the first 9 weeks of pregnancy [24, 25]. The second reason is the number of clinical patients in these two stages are relatively large and the data collection is relatively comprehensive. So, we collected the data at these two stages for this study.

Compared with previous study with higher AUC of estradiol in the 7th and 8th week, taking 590.5 pg/mL in the 7th week, and 614.5 pg/mL in the 8th week as cutoff levels of to estradiol predict bad pregnancy outcome [2], current results also showed that levels of estradiol individually or combination with progesterone has the higher AUC at 7-9 weeks. Our results taking 576 pg/ml as cutoff value levels of estradiol were slightly lower than 590.5 pg/mL in the 7th week, and 614.5 pg/mL in the 8th week in previous study. The difference may be our investigation interval was longer with 7-9 weeks. Current findings may help more women suffered miscarriage prior to 9 weeks than 8 weeks. Therefore, our study has certain rationality and advantages. Of course, our sample size is small, and larger samples and further prospective studies are needed to verify the results.

Supplementation with different progestogens in early pregnancy has been attempted to rescue a pregnancy in women with early pregnancy bleeding to treat threatened miscarriage [26]. Oral dydrogesterone are used to treat threatened abortion which was further stated effective in recent study [27]. In our study, oral dydrogesterone had no effect on serum progesterone levels, though higher levels of progesterone with giving dydrogesterone were shown than without starting oral dydrogesterone in two groups. It may be because dydrogestogen mainly alters maternal cytokine profiles to manage pregnancy complications [28]. Table 2 presented lower progesterone levels despite of no significance in women needed to be given oral dydrogesterone than without dydrogesterone. It may resulted from that women needed to be given oral dydrogesterone were diagnosed with threatened abortion in normal pregnancy group.

## Conclusions

Although ultrasound and continuous blood HCG value can, to a certain extent, determine the risk of miscarriage, it is still needed to predict high-risk pregnancy before clinical symptoms with other biomarkers [10]. Estrogen and progesterone reflecting the final pregnancy outcomes have been recognized, but few literature have indicated the specific hormone testing time and levels for miscarriage significance. In this study, we found that the miscarriage in first trimester may be predicted by estradiol levels, and the threshold sensitivity of cutoff value at 7–9 weeks of gestation is higher. Our study had limited cases and more case and multicenter studies need to be further researched.

Conclusively, low serum levels such as dual of estradiol and progesterone or estradiol alone at 7–9 weeks of gestation can be used to better predict miscarriage in first trimester. β-HCG or progesterone combing estradiol at 5–6 weeks of gestation may serve as better predictors also.

## Data Availability

Available from the corresponding author (dengwenhui@hotmail.com) and will be deposited in a public repository as soon as we gain the permission to do so.

## Abbreviations

β-HCG: β-human chorionic gonadotropin
ROC: Receiver operating characteristic
ART: Artificial assisted pregnancy technology
ICSI: Intracytoplasmic injection
IUI: Intrauterine insemination
AUC: Area under the ROC curve
PIBF: Progesterone induced blocking factor
